# Combining stool and stories: exploring antimicrobial resistance among a longitudinal cohort of international health students

**DOI:** 10.1186/s12879-021-06713-4

**Published:** 2021-09-27

**Authors:** Alena Kamenshchikova, Petra F. G. Wolffs, Christian J. P. A. Hoebe, John Penders, Hyun Y. Park, Mateus S. Kambale, Klasien Horstman

**Affiliations:** 1 Department of Health, Ethics and Society, School of Public Health and Primary Care (CAPHRI), Maastricht University, Postbus 616, 6200 MD Maastricht, The Netherlands; 2 Department of Medical Microbiology, School of Public Health and Primary Care (CAPHRI), Maastricht University Medical Centre (MUMC+), Maastricht, The Netherlands; 3 Department of Social Medicine and Medical Microbiology, School of Public Health and Primary Care (CAPHRI), Maastricht University, Maastricht, The Netherlands; 4 Department of Sexual Health, Infectious Diseases and Environmental Health, South Limburg Public Health Service (GGD South Limburg), Heerlen, The Netherlands; 5 School of Nutrition and Translational Research in Metabolism (NUTRIM), Maastricht University Medical Centre (MUMC+), Maastricht, The Netherlands; 6 Department of Pediatrics, the Netherlands School of Oncology and Developmental Biology, Maastricht University, Maastricht, The Netherlands; 7 Department of Clinical Research, Faculty of Infectious & Tropical Diseases, London School of Hygiene and Tropical Medicine (LSHTM), London, UK

**Keywords:** Antimicrobial resistance, Healthy travellers, CTX-M, NDM-1, India, Social science, Stories

## Abstract

**Background:**

Antimicrobial resistance (AMR) is a global public health concern that requires transdisciplinary and bio-social approaches. Despite the continuous calls for a transdisciplinary understanding of this problem, there is still a lack of such studies. While microbiology generates knowledge about the biomedical nature of bacteria, social science explores various social practices related to the acquisition and spread of these bacteria. However, the two fields remain disconnected in both methodological and conceptual levels. Focusing on the acquisition of multidrug resistance genes, encoding extended-spectrum betalactamases (CTX-M) and carbapenemases (NDM-1) among a travelling population of health students, this article proposes a methodology of ‘stool and stories’ that combines methods of microbiology and sociology, thus proposing a way forward to a collaborative understanding of AMR.

**Methods:**

A longitudinal study with 64 health students travelling to India was conducted in 2017. The study included multiple-choice questionnaires (n = 64); a collection of faecal swabs before travel (T0, n = 45), in the first week in India (T1, n = 44), the second week in India (T2, n = 41); and semi-structured interviews (n = 11). Stool samples were analysed by a targeted metagenomic approach. Data from semi-structured interviews were analysed using the method of thematic analysis.

**Results:**

The incidence of ESBL- and carbapenemase resistance genes significantly increased during travel indicating it as a potential risk; for CTX-M from 11% before travel to 78% during travel and for NDM-1 from 2% before travel to 11% during travel. The data from semi-structured interviews showed that participants considered AMR mainly in relation to individual antibiotic use or its presence in a clinical environment but not to travelling.

**Conclusion:**

The microbiological analysis confirmed previous research showing that international human mobility is a risk factor for AMR acquisition. However, sociological methods demonstrated that travellers understand AMR primarily as a clinical problem and do not connect it to travelling. These findings indicate an important gap in understanding AMR as a bio-social problem raising a question about the potential effectiveness of biologically driven AMR stewardship programs among travellers. Further development of the ‘stool and stories’ approach is important for a transdisciplinary basis of AMR stewardship.

**Supplementary Information:**

The online version contains supplementary material available at 10.1186/s12879-021-06713-4.

## Background

Antimicrobial resistance (AMR) is a growing global public health concern, which has been defined by its bio-social nature [1]. Although AMR is a biological phenomenon, the mechanisms leading to the development and dissemination of resistance genes are deeply social and include practices of antibiotic prescription and use [2, 3], practices of antibiotic production as well as waste management [4, 5]. Responding to this complexity, calls for transdisciplinary approaches to AMR have been formulated by various scholars in the field [6–8]. The Global Action Plan for AMR published in 2015 by the World Health Organisation (WHO) states that ‘everybody – in all sectors and disciplines – should be engaged’ in addressing this issue [7]. A policy brief from 2019 by the WHO emphasized the importance of cultural contexts for understanding and developing solutions for AMR [9]. Despite these calls, there has been a lack of studies that engage with both the biological and social nature of AMR simultaneously thus limiting the opportunities to address this problem from a transdisciplinary angle. To this end, in this article we explore a conceptual idea and present the results of transdisciplinary research that combines methods and concepts of microbiology and sociology, we called this methodology ‘stool and stories’. Stool and stories, we argue, should become a tool for transdisciplinary collaborations aiming at understanding AMR in its bio-social multiplicity.

To present the methodology of stool and stories, in this article we focus on the acquisition of multidrug resistance genes encoding the extended-spectrum beta-lactamase (ESBL) enzymes of the CTX-M group and the NDM-1 carbapenemases among a population of health students who travelled to India from different parts of the world. The mobility of healthy human populations has been shown to contribute to the dissemination of AMR globally [10, 11]. Microbiological research from the Netherlands showed that some international travellers have a high risk of acquiring AMR bacteria which persisted for up to 12 months after their return from a trip [12]. Multiple social, biomedical and environmental factors can increase the risk of acquiring resistant bacteria during travel, including antibiotic use, diarrhoea during travel, as well as eating behaviour, hygiene practices and overall sanitary conditions [12–15].

Applying the methodology of stool and stories to understand AMR and travelling we studied both incidence of AMR in a particular travellers’ group, and how these travellers give meaning to AMR. By making use of these different methods to understand AMR and travelling we aim to learn about potential linkages between the laboratory and social worlds of AMR. Learning about such linkages is crucial for a transdisciplinary understanding of AMR and the development of AMR stewardship programs that attune to diverse everyday practices.

## Methods

### Study participants

The study involved 64 people from a total cohort of 207 master students studying global health from a university in the Netherlands and a university in Canada of different nationalities. In 2017 these students were travelling from the Netherlands, Canada, Colombia, and Thailand to the south of India to participate in a two-week education symposium. The decision to focus on students was dictated by two important features of this travelling group: first, health students might be familiar with AMR and potential routes of its acquisition, which could be beneficial for our explorative research; and, second, due to the characteristics of their education program, students experienced multiple recent travels prior to this study.

### Study design

The stool and stories approach included three methods of data collection: laboratory analysis of faecal swabs samples, multiple-choice questionnaire (see Additional file 1), and semi-structured open interviews (see Additional file 1). To invite students to participate in the research, we provided all the study-related information to the potential participants and informed them about the use of anonymized microbiological data and the deidentification of interview data. Students could choose whether they were willing to participate in the whole study protocol, or only in one of the methods of data collection.

### Multiple-choice questionnaire

The aim of this method was to collect information about recent (3 months) travel history; history of antibiotic use during the previous year; health-related preparation for the trip to India; and demographic characteristics of the study group. The data were analysed using the software IBM SPSS Statistics Desktop Version 25.0. (IBM corp., Armonk, New York, USA). All 64 participants filled in the questionnaire. Most of the students filled in the questionnaire when they were in India (70%).

### Laboratory analysis of specimen

Of the 64 total participants, 45 participated in specimen collection. We provided participants with collection kits for faecal swabs, and this enabled them to collect samples themselves. The faecal samples were collected and stored in DNA/RNA shield (Zymo research, Irvine, USA) to ensure the stability of the metagenome during unrefrigerated transport and travel. Sampling time-points were: before travel (T0), during the first week in India (T1), and at the second week in India (T2). There was a small difference in participation for the faecal swabs at the moments in time. Before travel (T0), a total of 45 stool specimens were collected, from which 28 were collected in the Netherlands and 17 were collected directly at arrival to India for students coming from Colombia, Thailand, and Canada. During the first week in India (T1), 44 specimens were collected. In the second week in India (T2), 41 specimens were collected.

Faecal swabs were processed at the medical microbiology laboratory in the Netherlands after travel as follows: For the extraction of metagenomic DNA, 200 μL of the suspended faecal swabs were added to a 2 mL vial containing 0.5 g of 0.1 mm zirconia/silica beads (BioSpec, Bartlesville, OK, USA) and 1 mL of lysis buffer from QIAmp DNA stool kit (Qiagen). Samples were disrupted in a Magna Lyser device (Roche, Basel, Switzerland) in 3 cycles of 1 min. at 5,500 rpm. Subsequently, metagenomics DNA was isolated from the samples by using the QIAamp DNA stool kit according to the manufacturer’s instructions. DNA was eluted in 200 μL elution buffer and stored at − 20 °C until further analysis.

Real-time PCR was performed to detect and quantify the β-lactamase-encoding genes *bla*_CTX-M-1,_ *bla*_CTX-M-2_ and *bla*_CTX-M-9_ and *bla*_NDM_ (see Table 1). These genes were amplified using a 7900HT Fast Real-Time PCR Detection System (Applied Biosystems) in 25 μL reactions containing 12.5 μL Absolute QPCR ROX Mix (Thermo Scientific, Waltham, MA, USA) and 5-μL template DNA. The PCR was performed as described previously [16].

**Table 1:** PCR primer/probe sequences and additional PCR conditions to identify AMR genes in gut microbiota

Primer/probe	Sequence^a^ 5′ → 3′	Primer/probe concentration in reaction volume (nM)	Cycling conditions
CTX-M_Fw	ATGTGCAGYACCAGTAARGTKATGGC	500	95 °C, 15 min
CTX-M_Rv	ATCACKCGGRTCGCCNGGRAT	500	40 × 95 °C, 15 s;
CTX-M-1_probe	JOE-CCCGACAGCTGGGAGACGAAACGT-BHQ1	100	58 °C, 20 s
CTX-M-2_probe	6FAM-CAGGTGCTTATCGCTCTCGCTCTGTT-BHQ1	100	72 °C, 30 s
CTX-M-9_probe	JOE-CTGGATCGCACTGAACCTACGCTGA-BHQ1	100	
NDM_Fw	ATTAGCCGCTGCATTGAT	400	95 °C, 15 min
NDM_Rv	CATGTCGAGATAGGAAGTG	400	42 × 95 °C, 15 s;
NDM_probe	6FAM- CTG[+ C]CA[+ G]AC[+ A]TT[+ C]GGTGC-BHQ1	200	60 °C, 60 s

*nM* nanomolar

^a^Nucleic acids between brackets and preceded by + are locked nucleic acids

McNemar’s test for paired samples was used to compare resistance gene frequencies between the results from before and during travel. A p-value of < 0.05 (2-sided) was used as a significant cut-off. Statistical tests were performed by using IBM SPSS Statistics Desktop Version 25.0.

### Semi-structured interviews

The semi-structured interviews were conducted with 11 of the 64 participants to get a deeper understanding of how some of the participants viewed relationships between travelling, antibiotics, and AMR. In addition, we explored stories of participants about their health preparation for the trip to India, and precautionary health measures they thought were necessary before and during international travel. All interviews were conducted in April 2017, 2 interviews were conducted one week before the trip and 9 interviews were conducted within the first 2 weeks after arrival to India. The number of interviews was determined by data saturation. All interviews were conducted in person with the use of voice-recorder and transcribed verbatim. The analysis of the transcribed data was conducted in the NVivo 9 qualitative data analysis software with the use of thematic analysis (QSR International Pty Ltd, Doncaster, Victoria, Australia).

### Ethics

All students who participated in the study were registered and recruited through the university in the Netherlands. The study protocol was submitted to and approved by the local Medical Review Ethics Committee in the Netherlands. All participants signed informed consent forms. Although the students originated from partner universities in other geographic locations, ethical approvals from the partner universities were not required as all recruited participants were registered as students at the university in the Netherlands.

## Results

### Sharp increase in AMR genes after travelling

**Fig. 1:**
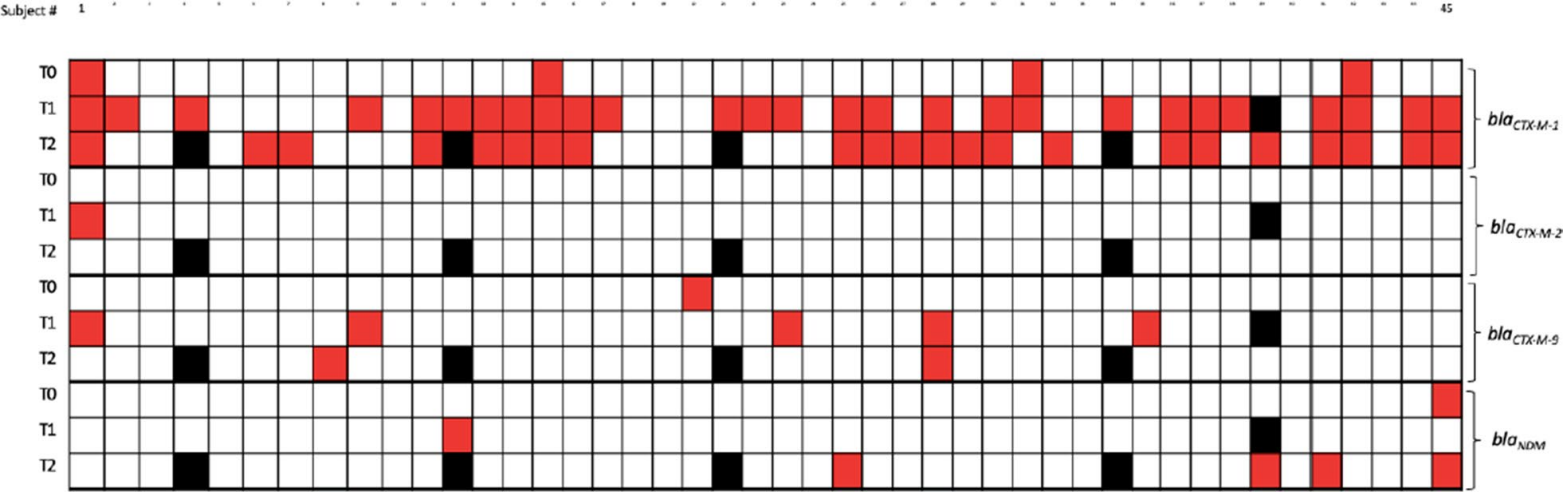
Participants positive for bla_CTX-M-1_, bla_CTX-M-2_, bla_CTX-M-9_ and bla_NDM_, genes detected in faecal metagenomics of 45 international travellers to India in three time-points (T0 = before, T1 = during, T2 = immediately after the travel) determined by qPCR. Data is represented in positive (red), negative (white), missing sample (black)

A total of 45 students participated in faecal sampling. Before travel, 5 of 45 (11%) students showed a faecal swab positive for *bla*_CTX-M_ genes, of which 4 swabs contained genes from *bla*_CTX-M-1_ group and 1 swab *bla*_CTX-M-9_ group. During the stay in India, 35 students tested positive for *bla*_CTX-M_ genes leading to a total positivity rate for these genes of 78%, which is a significant increase (p < 0.001). Thirty-two of 45 students (68%) acquired a new or additional type of *bla*_CTX-M_ gene. Of the 45 students, 40 were tested at both time-points during the trip (T1 and T2). Of this subset 27 acquired a new *bla*_CTX-M_ type during the trip and the majority: 13/27 (58%) were positive at both time-points, 6 only acquired a new *bla*_CTX-M_ type in T2 and 8 students acquired *bla*_CTX-M_ or an additional type of *bla*_CTX-M_ at T1, but lost them again at T2. For *bla*_NDM_, only 1 of 45 had a faecal swab positive for this gene prior to travel. An additional 4 students acquired *bla*_NDM_ during the stay and also this was a significant increase (p < 0.05) (please see Fig. 1).

### Global travellers and precautionary purchase of antibiotics

The analysis of the questionnaires showed that the median age of the participants was 27, and the majority of students were female (73%). The cohort of respondents represented a very mobile group, which was shown by their travelling practices. The majority (83%) of respondents visited more than 2 countries within the last three months before their arrival to India. The primary travel destinations were within Europe and included Belgium (37%), Germany (35%), and the Netherlands (30%), but also Canada (13%) and Thailand (10%). All of the participants visited India. These travelling trajectories can be explained by the international nature of the education program that students were involved in. Canada, Thailand and India were the three countries students could opt to go to for an elective. In addition, the proximity of the Netherlands to Belgium and Germany could also have contributed to the high students’ mobility between these countries.

Most of the participants did not take any antibiotics within the last year prior to the study (66%, n = 42), only a quarter of people took one course of antibiotics (25%, n = 16), and several people took 2 or 3 courses (9%, n = 6). Among those who did take antibiotics within the last year prior to the study, more than half was prescribed by a physician (59%, n = 13). However, 41% (n = 11) of those who used antibiotics within the last year informed us that they used non-prescribed antibiotics, which they got either from a family member or from a pharmacist without prescription in countries where it was possible.

Concerning their travel preparation to India, 14 out of 64 respondents reported that they brought antibiotics with them to the trip. The most prevalent antibiotics were azithromycin (42%, n = 5), amoxicillin (33%, n = 4), and ciprofloxacin (25%, n = 3). Two students indicated that it was important to take some antibiotics with them when travelling to India as a precautionary health measure.

### Travelling is not considered to be a risk for AMR

While microbiological methods analysed how international travelling may contribute to the acquisition of resistant bacteria, the method of semi-structured interviews was aimed at gaining a deeper contextual understanding of the daily practices that students exercised during their travel. Exploring the meanings of AMR, the interviewees often described this phenomenon in biomedical terms—as an ‘evolution of bacteria to resist antibiotics’. Such descriptions were not surprising as our participants had a background education in health. In addition, the interviewees were also participating in the collection of stool samples and the survey, and therefore they were previously exposed to information about AMR.

Building on a biomedical understanding of AMR our participants described several ways of how AMR can be developed and acquired. We distinguished two of such interrelated ways: first, AMR as an individual responsibility, and AMR as a medical phenomenon that can be acquired in clinical settings. This means that students associated mechanisms of AMR development with antibiotic use, and they connected AMR acquisition with clinical facilities.

In the interviews, students explained that the development of AMR is determined by individual use of antibiotics, including self-treatment, interruption of prescribed antibiotic treatment and its overuse. For instance, our participants explained,

Specific bacterial strains are becoming resistant to antibiotics because of the really often use of antibiotics (S21)

Antibiotic resistance is due to the fact that some people in many countries just taking many multi-resistant [antibiotics]. … The other reason is that many people don’t take the whole prescription that they’re supposed to take (S36)

Individual behaviour with respect to antibiotic use was defined by students as a major cause of AMR. In particular, the excessive amount of antibiotic use was associated with risks of resistance development.

Apart from the individual use of antibiotics, several students highlighted that environmental and infrastructural factors may play a role in AMR acquisition. Some students distinguished between a ‘risky country’ where antibiotics are used inappropriately, and a ‘risky environment’ like hospitals where AMR can be easily acquired by visitors and patients. However, these two definitions of ‘risky’ places are interrelated, as a respondent below explained that the risk of acquiring AMR is higher if a person gets admitted to a hospital in a particular country:

I know that especially some parts of the world, there is a lot of antibiotic use and because of this, this creates like bacteria that are already resistant to antibiotics and stay alive, and if this happened too much you create a resistance strain of antibiotics and I think this is becoming a more urgent issue. (S47)

So I am aware of different countries have different levels of antibiotic resistance. Probably if I get treated in a country where there is like a hospital where there is a lot of bacteria around that are resistant and I am exposed to this, then I might not be able to have them treated. (S20)

AMR, therefore, has been understood by our participants as rooted in individual practices of antibiotic use and in clinical practices of certain countries that create risks for AMR acquisition. These understandings were mirrored in students’ reflections on their health practices and travel preparations. Participants were surprised by our questions about the connection between AMR and travelling and they elaborated that they did not see such as a connection:

I always think about it [AMR] if I ever prescribed antibiotics and when I take it, not when I think of it when I am travelling or some kind, no, no, no. (S42)

While being aware of AMR, its biomedical nature and mechanisms, our participants argued that AMR as a problem was located in clinical settings rather than in public spaces. They did not associate and connect their international travelling practices with the potential risks of acquiring resistant bacteria unless they were admitted to a foreign hospital. This may indicate that while microbiological data highlight the importance of travellers’ social practices for understanding AMR, travellers themselves do not see AMR as a part of their social worlds but rather locate it in the clinical world.

## Discussion

In 2018, more than 6 billion people moved across the world, which included more than 4.3 billion air travellers [17], approximately 1.4 billion international tourists [18], as well as about 68.5 million forcibly displaced people [19]. Accompanying human travellers, bacteria are spread and disseminated to different parts of the world, which contributes to the global rise of AMR [14, 20, 21]. In this article, we attempted to develop a transdisciplinary understanding of this global rise of AMR by exploring the fruitfulness of a stool and stories methodological approach in a travelling group of international health students.

The different methods result in a different understanding of the relation between AMR and travelling. The microbiological findings from our study confirmed previous research [12, 16] pointing to the fact that travelling can be seen as a substantial risk factor for the acquisition of resistant bacteria. While overall microbiological data was not drastically different from other research with travelling groups, the acquisition of *bla*_NDM_ during the trip was higher as compared to other studies [12, 16]. We suggest that this can be partially explained by the high level of mobility among the study population, but likely also reflects the continuing dissemination and rise in carbapenem-resistant bacteria across the globe [22]. The data from the survey showed that international students are frequent travellers to various regions of the world. The surveys also showed that students anticipate the risk of specific diseases in different countries: some preventively purchase antibiotics in case they may fall ill. However, the analysis of the interviews showed that most participants did not relate travelling to AMR. They were explaining AMR by referring to antibiotic use of people or the presence of AMR in clinical settings in particular countries. The participants explained that as long as they were not using too many antibiotics and they were not hospitalised, they would not be exposed to AMR.

Our research had limitations. The sample size for all three methods was relatively small and the population was rather specific: health students who have prior knowledge of AMR. We understand that different results can be obtained from another group of travellers without an educational background in health. Moreover, the sample size for microbiological analysis was not equal at each moment as not all of the students who participated before travel (T0) were able to further participate in the study. However, this research demonstrates the potential fruitfulness of a transdisciplinary methodology of stool and stories.

**Fig. 2:**
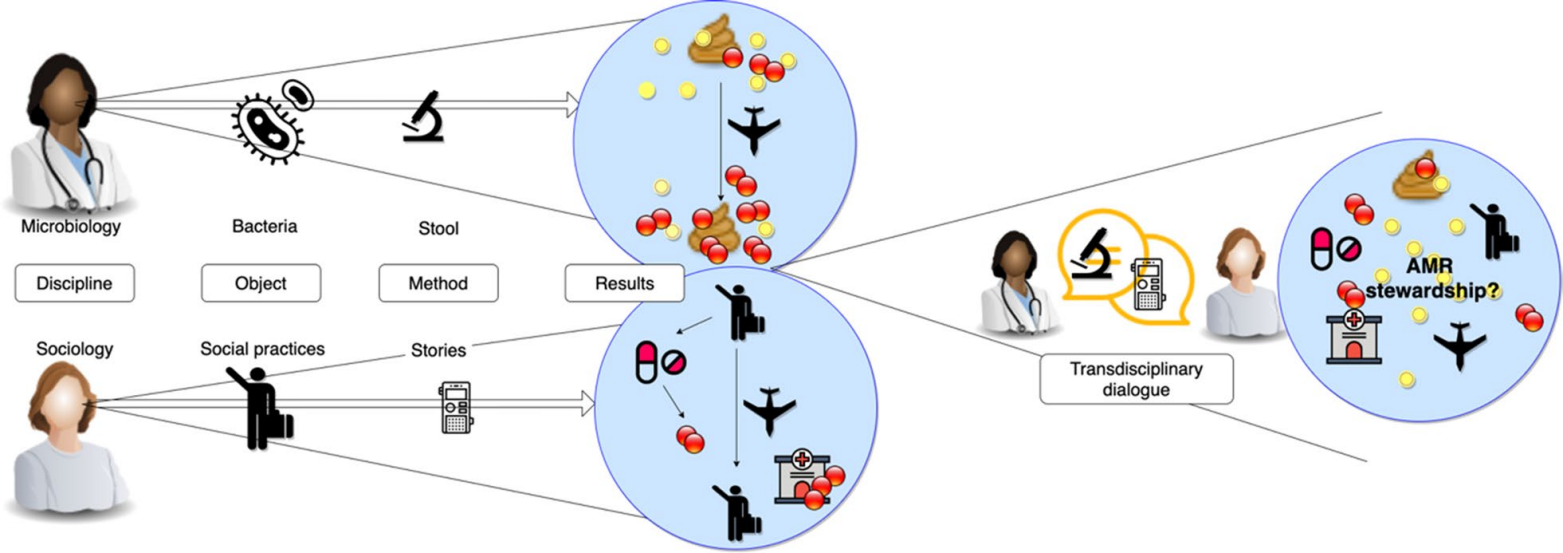
Although AMR has been an object of study for different disciplines, the methods, concepts, and therefore outcomes of different disciplinary analysis vary. Microbiologists are focusing on understanding bacterial communities and they use laboratory methods to understand how bacteria can develop resistance, how they can be acquired and disseminated. With relations to travelling, the methods of microbiology show that travelling to specific areas in the world can be understood as a potential risk factor for acquiring resistant bacteria. At the same time, sociologists study AMR by focusing on social communities and they apply methods such as interviewing and observation. With relations to travelling, sociological research shows that travellers perceive the potential risks of acquiring AMR only in a context of being admitted to a foreign hospital or if they over-use antibiotics, travelling in general is not considered to be a risk factor. Current disciplinary methods for understanding and conceptualising AMR run parallel to each other but are not in dialogue and cannot profit from each other’s expertise. Stool and stories is an invitation and a tool for such a dialogue

From the results, it becomes clear that the relations between AMR and travelling are understood differently by microbiological and sociological methods. While the analysis of stool showed how participants acquired resistant genes regardless of their antibiotic consumption; the analysis of the stories showed that participants constructed their understanding of AMR as a result of antibiotics use. Participants did not see travelling from one territory to another as a risk in itself (see Fig. 2).

This discrepancy in understanding of AMR sheds an interesting light on the current practices and policies related to antibiotic stewardship. The analysis of stool raises questions about the potential practices of travellers that could have contributed to their acquisition of resistant genes, like consumption of antibiotics, travellers’ diarrhoea and different food choices [12–15]. However, the sociological data showed that travellers were aware of AMR risks related to antibiotic consumption, and most of our participants reported that they did not consume or bring antibiotics with them to India. So, the stories of students challenged the presumed correlation between practices of individual travellers and their acquisition of AMR and stimulate the search for broader explanations of AMR acquisition, beyond the focus on individual antibiotic practices.

Reflecting on the results of sociological analysis, it is striking that the participants understanding regarding AMR is very much in line with international documents such as the Global Action Plan, a European One Health Action plan or the influential review on AMR developed by the British economist O’Neil, that highlight that it is the ‘systematic misuse and overuse’ of antibiotics that ‘put every nation at risk’ [7, 23, 24]. The underrepresentation of the relationship between AMR and travelling in international literature and policies is reproduced in the stories of the participants. It is the analysis of stool that shed a different light on stories—individual practices of antibiotic use might not be the key driver of participants acquisition of resistant genes and more attention should be given to broader environmental contexts within which the acquisition is taken place.

## Conclusions

Positioning stool and stories into a transdisciplinary dialogue challenges the current policy assumptions about AMR being primarily a problem of antibiotic consumption or a lack of antibiotic awareness. Rather, it may suggest a need for re-conceptualisation of AMR as a problem of environmental concern, broadening its current narrow focus on antibiotics and looking at various interactions between microorganisms, travellers, as well as local environments. This way AMR can become part of the world of travellers where some bacteria exist beyond the clinical settings and the world of travellers can become part of the laboratory where not all travelling practices are considered to be risky.

Without the integration of diverse perspectives on AMR, the health risks and preventive strategies highlighted by the laboratory can fail to be attuned to the social realities of travellers that may not see bacteria to be part of their daily life. We suggest that to improve the alignments of laboratory and societal practices, and to get more insight into the added value of transdisciplinary research, it is important to further develop the stool and stories approach. By bringing it into the dialogue and challenging the methodological and conceptual understandings of AMR in microbiology and sociology, stool and stories may help to create new and more systematic approaches to AMR.

## Supplementary Information

**Additional file 1.** Survey Questionnaire. Interview guide

## Data Availability

The data from this study is not available for open access but can be requested directly from the corresponding author.

## Abbreviations

AMR: Antimicrobial resistance
ESBL: Extended-spectrum beta-lactamases
WHO: World Health Organisation
