# Supplementary material for: Combining stool and stories: exploring antimicrobial resistance among a longitudinal cohort of international health students
Source: BMC Infect Dis. 2021 Sep 27;21:1008. doi: 10.1186/s12879-021-06713-4 (PMC8474840; doi:10.1186/s12879-021-06713-4)
Supplement: Supplementary file 1 — Additional file 1. Survey Questionnaire. Interview guide [file 12879_2021_6713_MOESM1_ESM.docx]

**Additional files**

***Additional file 1. Survey Questionnaire***

**Part I Biographical Information**

- Age: ……….

- Sex: M F

-Please circle one of the marital status: Married / Single / Divorced / Relationship

(Others, please specify:…………………………..)

-Citizenship: ………………………………………………………………………………………,

(If you have more than one citizenships, please indicate all.)

**Part II Questions (please mark the boxes □)**

1. Where are you currently living?

□ Maastricht/Netherlands □ McMaster/Canada □ Other (Specify)………………………………………………………

2. What is your eating habit?

□ Vegetarian □ Meat/Fish □ Mixed regimen

3. How many countries did you visit the last 3 months?

□1 □2-3 □4-5 □>5 □ None

4. Give the names of countries visited (if any and add the duration in days that you visited each country):

-

-

-

5. How many courses of antibiotics did you take in the last year?

□ 0, □ 1, □ 2-3, □ >3, □ I don’t know

6. If you took antibiotics in the last year, did you take antibiotic in the last 3 months?

□ Yes □ No

7. If yes, were they prescribed by a physician?

□ Yes □ No □ Both

8. If yes to question 5, for which reason did you take antibiotics?

□ Diarrheal syndrome

□ Respiratory complaints

□ Headache

□ Flu

□ Other (specify)……………………………………………………………………

10. Do you think it is important to take antibiotic before going to India for the Manipal Symposium?

□ Yes

□ No

□ I don’t know

11. Did you buy some antibiotic for your trip to Manipal?

□ Yes

□ No

12. If Yes, give the name of the antibiotic you have prepared for your trip …………………………………………………………………………………………………

13. If unknown, please indicate brief number of antibiotics ……………………………………………………………………………

14. Did you have diarrheal syndrome after an international trip the last 3 months?

□ Yes

□ No

15. (For people filling this survey while being in India), how often do you consume street food in India per week?

□ 1-2

□ 3-4

□ 4-5

□ >5

□ None

***Additional file 2. Interview guide***

*Introduction*

- Can you please introduce yourself and tell me about your trip to India? How it is going so far?
- Have you been to other countries before arriving in India? Which countries? Why did you go there? What did you like the most in different countries?
- Do you like to travel? Do you travel a lot? What are the usual reasons for you to travel?

*Health-related activities during travel*

- Have you ever experienced any health-related issues during your travelling, e.g. diarrhoea? What kind of issues? Can you give an example, maybe from one of your recent trips?
- What would you usually do if you feel some health-related discomfort during your travelling? Why? Do you prefer to go to local health facilities or do you carry some medicines with you? What kind of medicines?
- Do you have some health-related preparations before travelling, e.g. vaccination or preparation of some medicines? How do you decide on these preparation measures? Would it differ depending on the country you travel to? How?

*Antibiotic use*

- Do you take antibiotics with you when you go on a trip? Why? How do you usually buy antibiotics?
- What are your usual practices of antibiotic use? Do you take them for specific symptoms or after the doctor’s prescribing? How do you usually access antibiotic, do you need a prescription for these medicines in your home country?

*Antibiotic resistance and travelling*

- Have you ever heard about antibiotic or antimicrobial resistance? How would you explain it? What do you think can be the reasons for this phenomenon?
- Do you think there can be any relations between antimicrobial resistance and international travelling? Have you ever considered antibiotic resistance in relation to travels you make for your work, or your study travel or holidays?

*Travelling to India*

- Have you made any special health preparations before coming to India?
- Do you have some health routine here, or you just follow the same hygiene rules as in your home country?

*Reflection on study participation*

- How do you feel to be part of this research project? How did you feel about stool sample collection?
